# CRISPRi screening reveals *E. coli*’s anaerobic-like respiratory adaptations to gentamicin: membrane depolarization by CpxR

**DOI:** 10.1128/msystems.00353-25

**Published:** 2025-06-16

**Authors:** Donghui Choe, Eunju Lee, Yoseb Song, Sun Chang Kim, Ki Jun Jeong, Bernhard Palsson, Byung-Kwan Cho, Suhyung Cho

**Affiliations:** 1 Department of Bioengineering, University of California San Diego 8784, La Jolla, California, USA; 2 Department of Biological Sciences, Korea Advanced Institute of Science and Technology, Daejeon, South Korea; 3 KI for the BioCentury, Korea Advanced Institute of Science and Technology, Daejeon, South Korea; 4 Department of Chemical and Biomolecular Engineering, Korea Advanced Institute of Science and Technology, Daejeon, South Korea; 5 Graduate School of Engineering Biology, Korea Advanced Institute of Science and Technology, Daejeon, South Korea; 6 Department of Pediatrics, University of California San Diego 8784, La Jolla, California, USA; University of Minnesota Twin Cities, Minneapolis, Minnesota, USA

**Keywords:** CRISPR interference, gene essentiality, antibiotics, resistance, CpxR

## Abstract

Bacterial genes serve diverse cellular functions and many affect fitness in response to environmental challenges. We employed CRISPR interference screening to investigate the fitness effect of each gene in *Escherichia coli* exposed to gentamicin, aiming to understand the cellular defense mechanisms. Our findings revealed that ribosomal proteins, ribosome-associated proteins, toxin-antitoxin systems, and outer membrane proteins strongly influence the fitness of *E. coli* in gentamicin. Notably, gentamicin-induced fitness changes resembled those under anaerobic conditions, where resistance to gentamicin was observed. Specifically, genes related to the biosynthesis of cofactors and electron carriers, crucial for the respiratory system, showed reduced essentiality under both gentamicin and anaerobic conditions, suggesting a disruption in membrane potential leading to limited gentamicin uptake. Transcriptomic and genome-wide binding analyses identified the two-component system CpxR as a key regulator of respiratory systems in response to gentamicin. Our study provides insights into cellular defense mechanisms, offering potential strategies for combating antibiotic resistance.

**IMPORTANCE:**

Bacteria can adapt to a variety of stressful environments, including antibiotic exposure. The mechanisms underlying antibiotic resistance remain an active area of investigation. Clustered regularly interspaced short palindromic repeats (CRISPR) interference enables specific silencing of gene expression, allowing researchers to assess the fitness effects of gene knockdowns under given conditions. Using genome-wide CRISPR interference screening on *Escherichia coli* exposed to gentamicin, we identified anaerobic-like fitness effects of genes involved in respiration and the maintenance of membrane potential—key processes that facilitate gentamicin entrance into the cell. Transcriptomic analysis and immunoprecipitation assays further indicated that the two-component system CpxR modulates respiratory adaptations in response to gentamicin challenge. These findings shed light on the development of antibiotic resistance in bacteria and may offer new insight into strategies for treating gentamicin-resistant pathogens.

## INTRODUCTION

Bacteria adapt to environmental conditions through genetic modifications, transcriptional and translational regulation, enabling survival in diverse and often hostile environments. Genetic changes induce different fitness effects to environmental stresses, such as antibiotics, resulting in expansion and extinction of certain bacterial species. Thus, investigating gene fitness is crucial for uncovering the development of defense and resistance mechanisms against antibiotics. Conventional methods for examining gene essentiality, such as gene knockout or site-directed mutagenesis using oligonucleotides (1) and the transposon-driven gene disruption (2), have focused on observing cell death. While these methods contribute to identify genes essential for survival, they often fail to quantitatively assess the fitness effects of essential genes due to the lethality caused by complete loss of gene. However, the advent of CRISPR interference (CRISPRi) technology enables us to identify the genes required for cell survival without causing cell death (3, 4). The CRISPR-Cas9 system has been applied to a wide range of applications (5) and shown to offer minimal off-target effects, low noise, and greater consistency compared to shRNA-based systems for gene fitness screens (6).

In this study, we focused on genome-wide fitness changes induced by gentamicin exposure. Gentamicin, a member of the aminoglycoside antibiotic class, is widely used to treat various infections, including septicemia, meningitis, and urinary tract infections (7). Aminoglycosides are bactericidal antibiotics with a broad spectrum of activity, targeting aerobic gram-negative bacteria, including Enterobacteriaceae, and certain gram-positive bacteria (8). These antibiotics enter cells through both the outer and inner membranes, subsequently inhibiting translation by targeting the ribosome. This inhibition leads to protein mistranslation by incorporating incorrect amino acids during protein elongation (9, 10). The resulting misfolded proteins are incorporated into the cell envelope, destabilizing the membrane and causing oxidative stress, which ultimately leads to cell death (11, 12).

Given the multifaceted actions of gentamicin, bacterial cells undergo fitness changes to evade its toxic effects. For instance, membrane depolarization may reduce antibiotic uptake or increase efflux activity, both of which lower intracellular aminoglycoside concentrations and reduce binding to ribosomal RNA. Such resistance mechanisms may include aminoglycoside-modifying/degradation enzymes, efflux systems, sequestrating proteins, target modification/bypass proteins, and mobile genetic elements (13–15).

However, these resistance mechanisms do not act uniformly across all bacterial strains. The mode of action depends on both the specific antibiotics and the bacterial species involved. Gentamicin is known primarily as a translation inhibitor, but its complete mode of action remains to be understood. Additionally, gentamicin is ineffective under anaerobic conditions, as it fails to enter *Escherichia coli* cells without oxygen (16). This inefficiency also explains why certain aminoglycosides, including gentamicin, are less effective when administered orally, given the anaerobic conditions in the intestines. Understanding the lack of function in anaerobic environments may shed light on the mechanisms underlying antibiotic resistance. Therefore, we also examined the fitness changes of *E. coli* under anaerobic conditions to gain further insight into resistance mechanisms against gentamicin. Using a high-resolution CRISPRi library, we identified gentamicin-sensitive genes on a genome-wide scale and explored their roles in resistance. Furthermore, transcriptomic analysis using iModulonDB and binding studies revealed that the response regulator CpxR plays a significant role in gentamicin resistance.

## RESULTS

### Genome-wide gene fitness analysis using CRISPRi library screening

**Fig 1:**
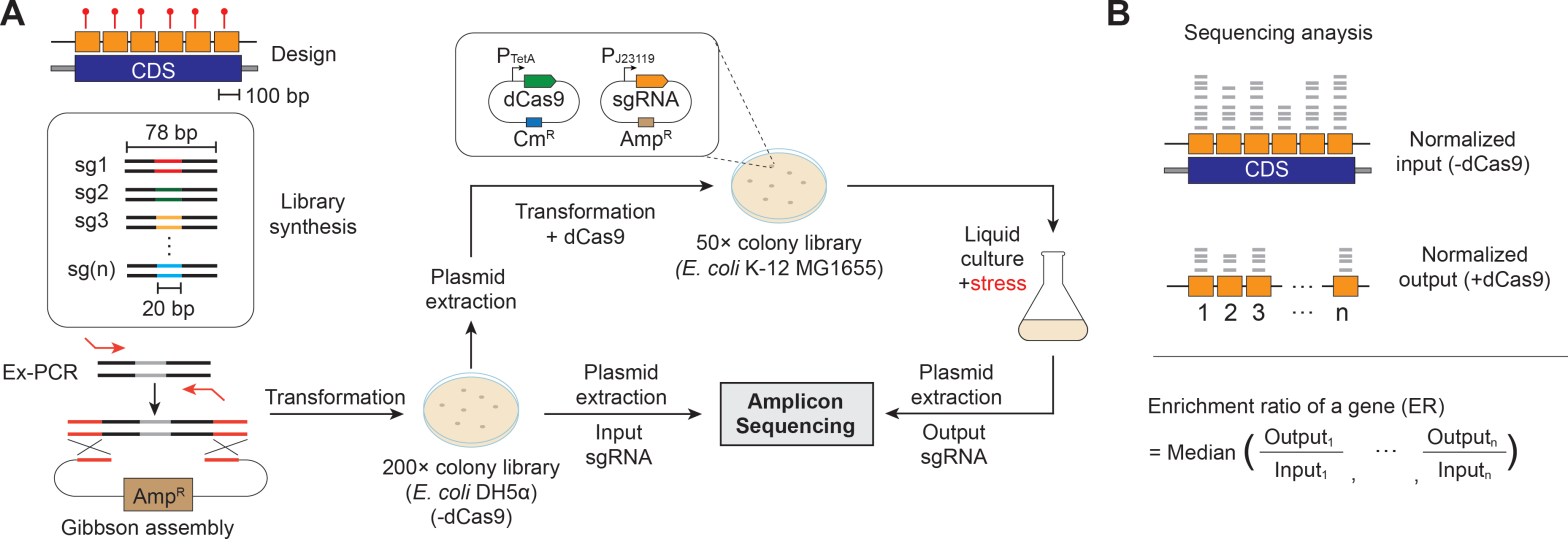
Genome-scale CRISPRi screen. (A) Schematic of the CRISPRi screening process. A genome-wide sgRNA library was designed to target every 100 bp of coding sequences, synthesized, and constructed. The sgRNA library plasmids were first transformed into *E. coli* DH5a to create an initial plasmid library. These plasmids were then extracted and transformed into *E. coli* K-12 MG1655 carrying the dCas9 plasmid. Cells were cultured in LB liquid media under various stress conditions, and after harvesting, the sgRNA populations were sequenced using next-generation sequencing (NGS) following amplicon amplification. (B) Library analysis. The enrichment ratio (ER) for each gene was defined as the median of the normalized sgRNA ratios across the positions targeted in the gene.

We utilized the CRISPRi system to probe the effect of gentamicin on gene fitness in *E. coli*. Most CRISPRi library designs focus on targeting a single or a few positions within a gene. However, CRISPRi efficiency can vary depending on the genomic structure or interactions between the dCas9 complex and DNA. Even if a specific position is efficient for CRISPRi activity, it is difficult to ensure consistent efficiency under all environmental conditions. To overcome this, we designed a high-resolution genome-scale CRISPRi library targeting the protospacer adjacent motif (PAM) sequences at intervals of 100 bp across all coding sequences (CDS) as described in Materials and Methods (Fig. 1A). We synthesized sgRNA library targeting 39,591 positions, covering all CDS of *E. coli* in the form of an oligonucleotide pool. The pools were cloned into an expression plasmid via extension PCR and Gibson assembly (Fig. 1A). Following plasmid transformation into *E. coli* DH5α without dCas9 co-expression, we collected approximately 8 × 10^6^ colonies on LB agar plates, equivalent to 200 × coverage of the pool. The colonies were pooled and cultured in liquid LB media to an OD 1.0, after which plasmids were extracted for amplicon sequencing. We confirmed that the initial library included 39,574 sgRNAs, representing 99.96% coverage (Table S1).

Next, the plasmid library was transformed into *E. coli* K-12 MG1655 expressing dCas9, and approximately 2 × 10^6^ colonies were collected from LB agar, ensuring a 50-fold coverage of the sgRNA pool. These colonies were then cultured in liquid LB media, either untreated or treated with gentamicin. Based on the dose-response curve (Fig. S1), gentamicin was used at a sub-lethal concentration of 1 µg/mL, which has been reported to induce resistance and stress responses, including biofilm formation in *E. coli* (17). We then analyzed the abundance of sgRNAs in pooled cells, with and without dCas9 co-expression (Table S2). To assess the fitness effects of each sgRNA, we calculated the enrichment ratio (ER) by comparing the abundance of sgRNAs with dCas9 co-expression to that in the initial pool (without dCas9). The ER for each gene was defined as the median ratio of all sgRNAs targeting that gene (Fig. 1B; Table S3). An ER of 1 indicates that CRISPRi-mediated knockdown had no effect on fitness, whereas an ER below 1 suggests a detrimental effect, and an ER over 1 suggests a beneficial effect for cell growth.

### Fitness changes of ribosome and ribosome-associated proteins in response to gentamicin

**Fig 2:**
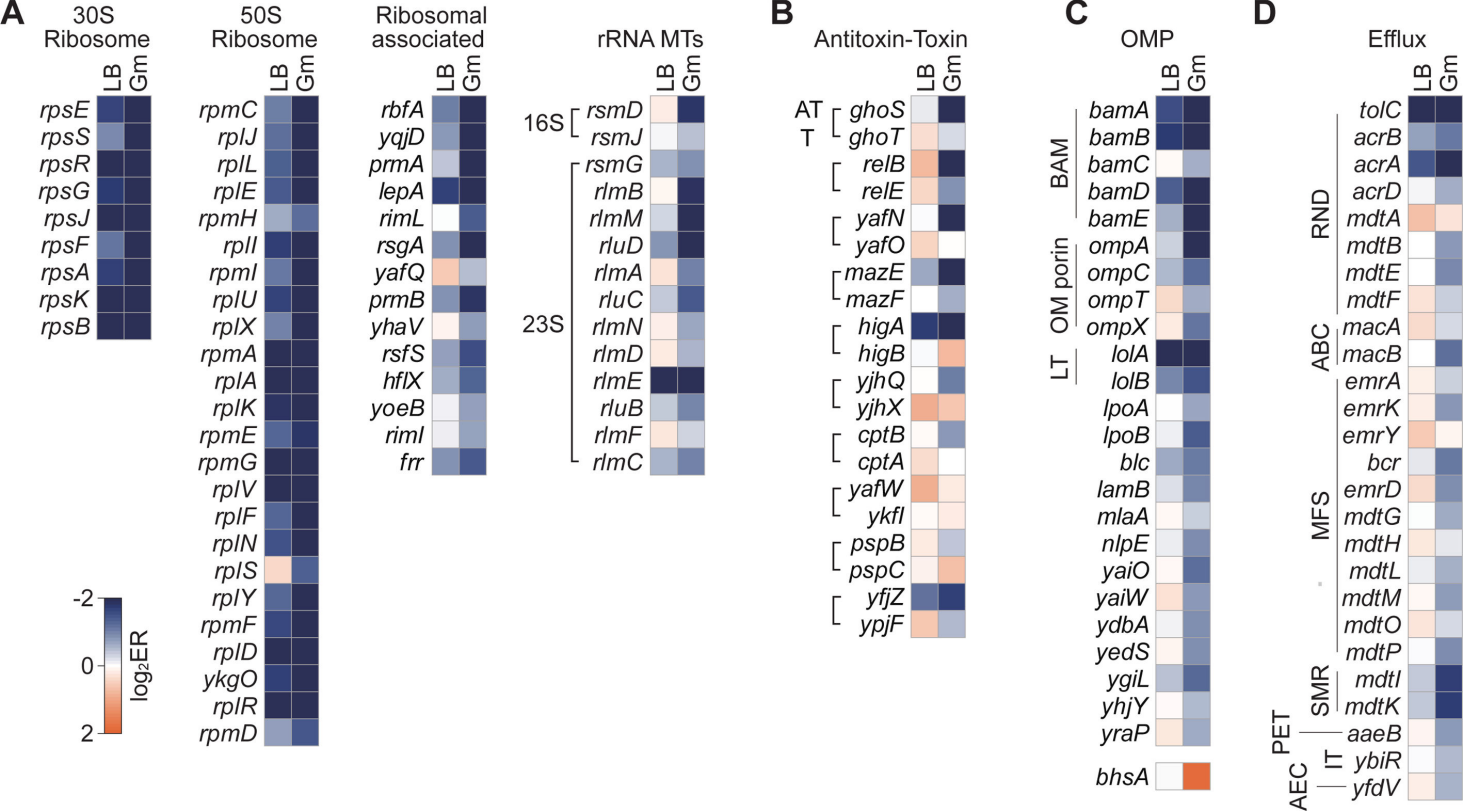
Gene enrichment ratio (ER) in response to gentamicin treatment. (A) Genes encoding 30S ribosomal subunits, 50S ribosomal subunits, ribosome-associated proteins, and rRNA methyltransferases (MTs) with an ER fold change (ER_LB_/ER_Gm_) of 1.5 or higher are listed in descending order based on ER value. (B–D) Genes associated with antitoxin-toxin systems (B), outer membrane proteins (OMP) (C), and efflux systems (D) showing an ER fold change of 1.5 or higher are similarly listed in descending order. An exception is noted for *bhsA* (in the OMP category), which exhibited the highest ER under gentamicin, with a reverse fold change (ER_Gm_/ER_LB_) of 3.8. AT: antitoxin. T: toxin. BAM: β-barrel assembly machinery. LT: lipoprotein trafficking pathway. RND: resistance-nodulation-division family transporter. ABC: ATP-binding cassette transporter. MFS: major facilitator superfamily transporter. SMR: small multidrug resistance family transporter. PET: putative efflux transport (PET) family transporter. IT: ion transporter superfamily transporter. AEC: auxin efflux carrier family transporter.

Gentamicin is an antibiotic that exerts its effect by binding to the 30S subunit of bacterial ribosomes, leading to translation inhibition. To investigate the impact of gentamicin on ribosome fitness, we examined the fitness changes of ribosome, ribosome-associated proteins, and ribosomal RNA (rRNA) modification proteins. Since ribosomes are essential for cellular function, most 30S and 50S ribosomal proteins showed low ERs in LB media (Fig. 2A). However, after gentamicin treatment, the ERs of many ribosomal proteins were significantly decreased, indicating the sensitivity of ribosomes to gentamicin. Among genes encoding ribosome-associated proteins, *rbfA*, whose protein product binds the 30S subunit and is involved in 16S rRNA processing (18), showed a 5.0-fold decrease in ER under gentamicin treatment.

Additionally, the gene encoding YqjD, an inner membrane protein associated with the 30S subunit, exhibited a 4.7-fold ER reduction. Other notable reductions included *prmA* encoding a ribosomal methyltransferase (3.2-fold), *lepA* encoding a 30S ribosome biogenesis-associated protein (2.9-fold), *rimL* encoding a ribosomal N-acetyl transferase known to confer resistance to microcin C (2.7-fold) (19), and *rsgA* encoding a 30S subunit-associated GTPase that is inhibited by aminoglycoside (2.2-fold). Ribosomal modification through acetylation, adenylation, or phosphorylation can alter the target site of aminoglycosides, thereby inactivating antibiotic binding. We further investigated the fitness change in rRNA-modifying proteins. In previous studies, methylation in the 16S rRNA at residue A1408 or G1405 by 16S rRNA methyltransferases was identified as a mechanism of resistance to aminoglycoside (20). Among the rRNA-modifying proteins, genes encoding 16S rRNA methyltransferase, *rsmD* (4.1-fold), 23S rRNA methyltransferases such as *rlmB* (4.0-fold), *rlmM* (3.5-fold), and *rlmA* (2.5-fold), as well as 23S rRNA pseudouridine synthases *rluD* (3.1-fold) and *rluC* (2.1-fold), showed significantly reduced ERs. Taken together, we identified key ribosomal proteins, ribosome-associated proteins, and rRNA-modifying proteins that exhibit increased sensitivity to the aminoglycoside gentamicin.

### Fitness changes of toxin-antitoxin systems in response to gentamicin

We next investigated the fitness changes in bacterial toxin-antitoxin (TA) systems, which play a critical role in the development of antibiotic resistance and persistence (21). Under gentamicin treatment, several TA systems, including *ghoST*, *relBE*, *yafNO*, *mazEF*, and *higBA*, showed significant fitness reductions, with ERs more than 2-fold lower compared to the LB control (Fig. 2B).

According to the widely accepted hypothesis that toxins contribute to persistence under stress (22), inhibition of the toxin using CRISPRi led to a low ER, which is consistent with the belief that toxin activity is important for survival under gentamicin exposure. However, the inhibition of antitoxin genes, which theoretically should activate the toxin and promote persistence, did not result in increased ER. Instead, we observed a much lower ER, indicating that unregulated toxin activity resulting from antitoxin inhibition caused significant cellular damage (Fig. 2B).

In the GhoST system, *ghoT* encodes the toxin of a type V system, which causes membrane damage, reduces cellular ATP levels, and disrupts the proton motive force (PMF), impacting membrane potential (23, 24). In contrast, *ghoS*, the antitoxin, neutralizes the toxin by specifically cleaving the *ghoT* coding region in the *ghoST* transcript (24). While *ghoT* showed a 1.5-fold decrease in ER under gentamicin, *ghoS* showed a dramatic 9.8-fold decrease, indicating that the antitoxin activity is crucial for mitigating the membrane damage caused by gentamicin. The low ER for *ghoS* supports the hypothesis that, under gentamicin stress, the antitoxin plays a more complex regulatory role. While the toxin can promote stress adaptation or persistence, inhibiting the antitoxin leads to uncontrolled toxin activity, resulting in severe cellular damage. This explains the greater fitness cost observed when inhibiting antitoxin genes, as the loss of regulatory control over the toxin causes unregulated membrane damage and worsened fitness.

RelE, a toxin that cleaves mRNA at the ribosomal A site (25), exhibited a 2.5-fold decrease in ER, whereas its antitoxin RelB showed a 7.0-fold decrease. This suggests that RelB strongly controls RelE during gentamicin exposure. Similarly, YafN, an antitoxin of the ribosome-dependent mRNA interferase YafO that inhibits translation (26), showed a 6.7-fold decrease in ER. The gene encoding MazE antitoxin, known to form a complex with the MazF sequence-specific ribonuclease that causes programmed cell death in response to antibiotics such as rifampicin, chloramphenicol, and spectinomycin (27), exhibited a 3.2-fold decreased ER. *higA*, encoding an antitoxin against HigB, a ribosome-dependent mRNA interferase toxin that inhibits protein synthesis by cleaving translated mRNAs (28), showed a 3.0-fold decreased ER.

Moreover, antitoxins may also play regulatory roles beyond simply neutralizing the toxin. Antitoxins, alone or in complex with their cognate toxin, can help maintain a balance between various cellular processes in response to stress (29–31). Inhibiting antitoxin genes results in the loss of regulatory control, which exacerbates cellular stress and causes unregulated damage to the cell, ultimately reducing fitness.

Additionally, many toxin-antitoxin systems are co-transcribed in operons, such that the inhibition of the upstream antitoxin could potentially have a polar effect on the downstream toxin gene (*ghoST*, *relBE*, *yafNO*, *mazEF*, and *cptBA*). This would result in the inhibition of both genes simultaneously, rendering no significant toxin activation. However, we did not observe such polar effects in the TA systems (Fig. 2B). This absence of polar effects can likely be attributed to the important regulatory roles of the antitoxins, which go beyond simply neutralizing the toxin.

In summary, these findings highlight the dual roles of both toxins and antitoxins in bacterial survival under gentamicin stress. While toxins contribute to the stress response and adaptation to antibiotic challenge, their activity must be tightly regulated by antitoxins to prevent excessive cellular damage.

### Fitness changes of outer membrane proteins and efflux pumps upon gentamicin

Next, we investigated the fitness effects of outer membrane proteins (OMPs), which are associated with basic physiological functions, virulence, and multidrug resistance. OMPs play a fundamental role in maintaining cellular viability (32). Among genes encoding OMPs, *ompA* exhibited the greatest abundance change under gentamicin, with a 3.8-fold ER decrease (Fig. 2C). OmpA is a major outer membrane protein that maintains membrane integrity but has lower permeability compared to other porins (33). Interestingly, studies have shown that *ompA* deletion increases susceptibility to several antibiotics, including β-lactams, in *Acinetobacter baumannii (*34).

The ER of *bamE*, which encodes a protein involved in assembling OmpA and forming a complex with BamABCD, was also highly decreased by 2.6-fold. The *bamE* deletion strain is known to increase sensitivity to rifampicin, cholate, and SDS/EDTA (35), and it may have a similar effect under gentamicin treatment. Additionally, the ER of *nlpE*, encoding a sensor lipoprotein that interacts with OmpA and activates the Cpx signaling pathway, decreased by 1.8-fold. Despite the expectation that cells with porin knockdown would be enriched as porins may facilitate gentamicin uptake, most porins showed decreased or unchanged ERs, except for *bhsA* (Fig. 2C). This suggests that the role of porins in nutrient uptake and preventing membrane damage outweighs their role in gentamicin uptake.

We also investigated the fitness of efflux pumps, which protect cells from various stresses and chemicals introduced through transporters or porins (36, 37). Activation of the efflux system is a key mechanism of aminoglycoside resistance (Fig. 2D). We found significant reductions in the ERs of several efflux pump genes. *tolC*, which encodes a common outer membrane channel for RND (resistance-nodulation-division family) transporters, ABC transporters, and MFS proteins that pump out toxic chemicals (38), showed a 1.5-fold ER decrease. The RND transporter genes, *acrA*, *mdtB*, and *mdtE*, showed 2.6-, 1.9-, and 2.0-fold decreases in ER, respectively. ABC transporter genes, *macA* and *macB*, also showed significant ER decreases (1.6- and 2.3-fold, respectively), as did MFS transporter genes, *emrK*, *bcr*, *merD*, *mdtM*, and *mdtP*, which had 1.8- to 2.5-fold decreases. The small multidrug resistance (SMR) family gene, *mdtI,* and the MATE family gene, *mdtK*, showed 2.6-fold decreases as well. These findings suggest that efflux systems may play a significant role in gentamicin resistance.

### Similar fitness changes in anaerobiosis and gentamicin treatment

**Fig 3:**
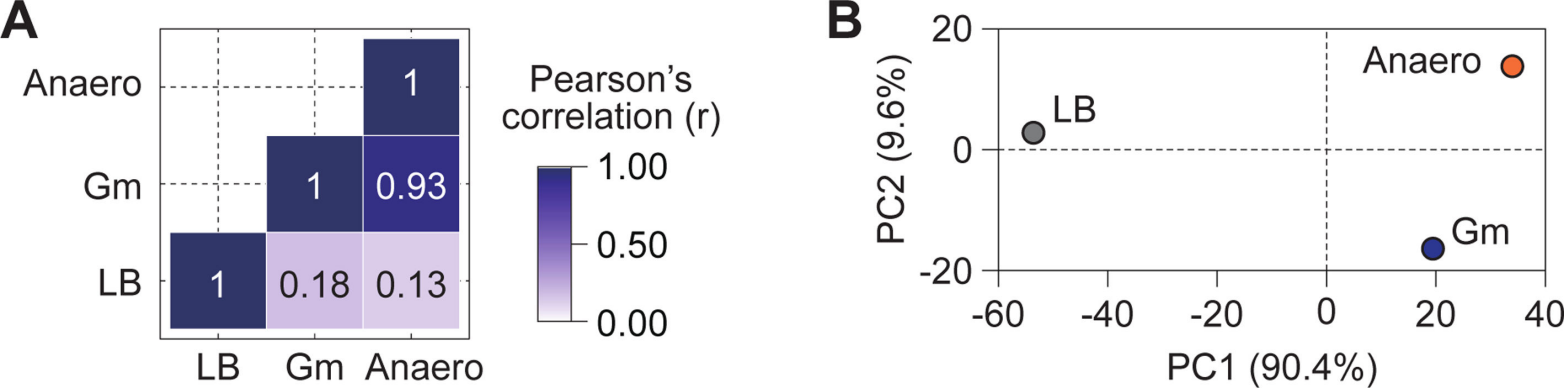
Similarities in fitness changes under gentamicin and anaerobic conditions. (A and B) Pearson correlation coefficient analysis (A) and principal component analysis (PCA) (B) were performed to compare fitness changes across different conditions: LB medium without supplementation, LB with gentamicin, and anaerobic conditions, based on ER values.

Aminoglycosides enter the periplasmic space through passive diffusion via outer membrane porins (39). However, their transport into the cytoplasm requires metabolic energy provided by the respiratory/electron transport system (40), suggesting that aminoglycoside uptake is blocked under anaerobic conditions. Consistent with this, we also found that *E. coli* was completely resistant to gentamicin under anaerobic conditions (Fig. S2). To understand the impact of anaerobiosis on gentamicin resistance, we analyzed fitness changes induced by gene knockdowns using CRISPRi screening. Pearson correlation coefficient (*r*) analysis revealed a strong correlation between the ER ratio of genes under anaerobic conditions and gentamicin treatment (*r* = 0.93) despite the different types of stress (Fig. 3A). PCA analysis further supported this strong correlation, showing that, while anaerobic and gentamicin conditions are slightly separated along principal component 2, they are closely aligned along principal component 1, which explains the majority of the variance (Fig. 3B).

**Fig 4:**
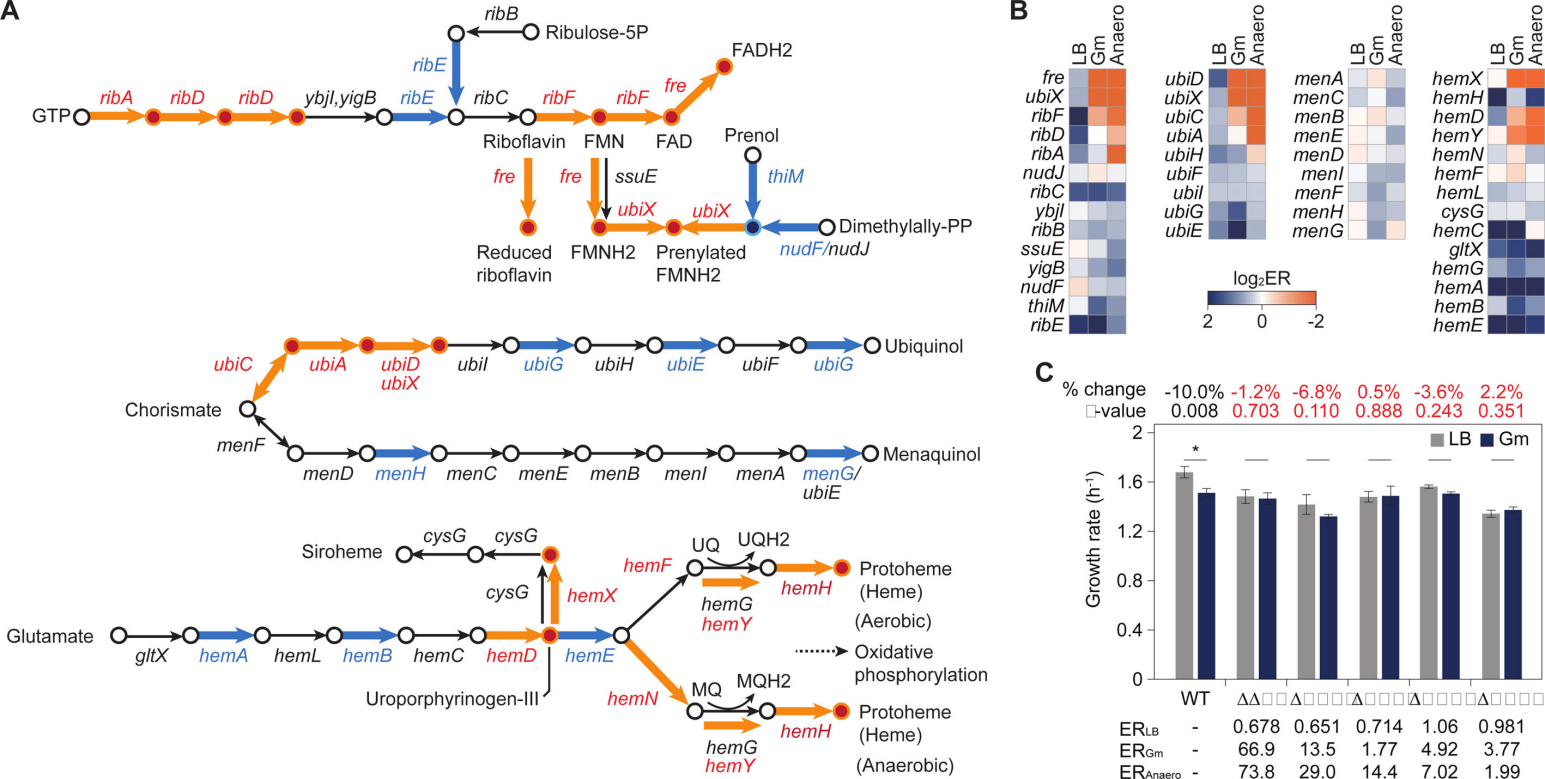
Fitness changes of genes involved in coenzyme transport and metabolism under gentamicin treatment. (A) Pathways associated with riboflavin, quinone, and heme biosynthesis are displayed. Arrows in orange color indicate genes with an ER increase of 1.5-fold or more under gentamicin, while arrows in blue color indicate genes with an ER decrease of 1.5-fold or more. (B) Heatmap showing the ERs of genes involved in the pathways shown in panel A under LB medium, gentamicin (Gm), and anaerobic (Anaero) conditions. (C) Growth rate of *E. coli* BW25113 single-knockout strains of cofactor biosynthetic genes. Error bars indicate the standard deviation (SD) of three biological replicates. Knockout mutants resistant to gentamicin are shown in red. *P-value < 0.1 (Welch's *t*-test).

To analyze the function of genes affected by gentamicin or anaerobic conditions, we examined genes with more than a 1.5-fold change in ER compared with LB conditions. Clusters of Orthologous Groups (COG)-based functional categorization (41) revealed that knockdown of genes related to coenzyme transport and metabolism showed beneficial effects, with significantly increased ER under both stresses (Fig. S3). Among those, the riboflavin, quinone, and heme biosynthesis pathways were particularly affected (Fig. 4A and B).

In the riboflavin biosynthesis pathway, genes such as *ribA* (1.6-fold under gentamicin and 8.6-fold under anaerobic conditions), *ribD* (3.0- and 5.4-fold), *ribF* (9.4- and 17.0-fold), *fre* (67.0- and 73.8-fold), and *ubiX* (13.5- and 30.0-fold) exhibited significant ER increases under both gentamicin and anaerobic conditions. RibA and RibD catalyze the early steps of riboflavin biosynthesis, and their essentiality decreased under these conditions. In addition, *ribF*, which transforms riboflavin into the coenzymes FMN and FAD, and *fre*, a flavin reductase that reduces free flavins, riboflavin, FMN, or FAD by NADPH or NADH, both showed substantial ER increases.

In the quinone synthesis pathway, *ubiC* (2.5- and 20.2-fold), which catalyzes the first committed step in ubiquinone biosynthesis, and *ubiA* (1.6- and 8.1-fold), *ubiD* (30.4- and 45.6-fold), and *ubiX* (20.7- and 44.5-fold), which catalyze later steps, also showed markedly increased ER under gentamicin and anaerobic conditions (Fig. 4A and B). Mutants lacking *ubiA*, *ubiD*, and *ubiE*/*ubiG* are known to be impaired under anaerobic conditions (42). Interestingly, some genes involved in menaquinol biosynthesis showed decreased ER under gentamicin and anaerobic conditions, reflecting the differing electron transport preferences—ubiquinone during aerobic growth and menaquinone during anaerobic conditions. While we did not directly analyze the electron transport chain in this study, the observed fitness changes under both conditions suggest similar impacts, consistent with the idea that gentamicin treatment induces effects that are highly similar to anaerobic metabolism, especially in relation to membrane potential.

Heme biosynthesis genes also exhibited increased ER under both gentamicin and anaerobic conditions, including *hemD* (3.8- and 7.6-fold), *hemX* (4.7- and 6.6-fold), *hemY* (2.8- and 4.8-fold), and *hemH* (4.0- and 1.6-fold). As a critical cofactor in processes such as respiration and detoxification, heme appears to play a role in gentamicin resistance.

Deletion strains of key cofactor biosynthesis genes, including *fre*, *ubiX*, *ubiC, and hemX*, exhibited less sensitivity to gentamicin compared to wild-type strains (Fig. 4C). This aligns with a previous study showing that a ubiquinone-deficient mutant had reduced aminoglycoside uptake due to a lowered membrane potential, which is necessary for gentamicin uptake (43). Deletion of *bhsA*, encoding a stress-resistant outer membrane protein that increases hydrophobicity (44), conferred complete gentamicin resistance (Fig. 4C). The *bhsA* deletion prevents polycationic aminoglycoside permeabilization (39), consistent with the 3.8- and 2.0-fold ER increases seen in CRISPRi studies under gentamicin and anaerobic conditions, respectively.

Taken together, the fitness changes induced by gentamicin are highly similar to those seen in anaerobic metabolism, particularly in the context of cofactor biosynthesis and its related genes. These findings suggest that gentamicin resistance may be linked to metabolic shifts affecting membrane potential through altered electron transfer.

### Regulation of respiratory complexes by CpxR and ArcA, resulting in membrane depolarization

**Fig 5:**
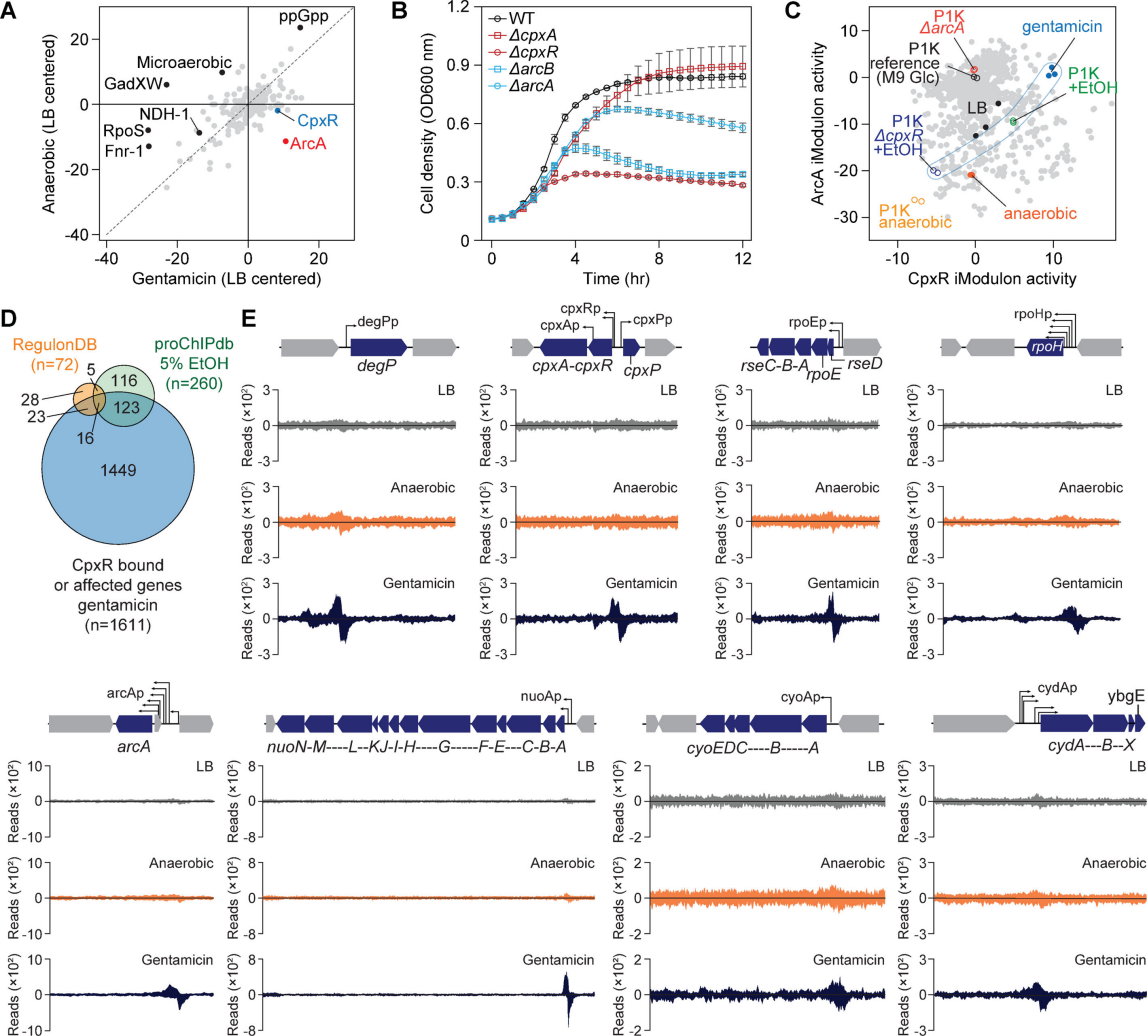
Transcriptome and genome-wide binding analyses of CpxR and ArcA under anaerobic and gentamicin stresses. (A) Differential iModulon activity between anaerobic and gentamicin stresses. Each dot represents an individual iModulon. (B) Growth profiles of *arcA*, *arcB*, *cpxA*, and *cpxR* knockout strains under gentamicin treatment (1 µg/mL). (C) CpxR and ArcA iModulon activities of *E. coli* under anaerobic and gentamicin stresses. Gray dots indicate over 1,000 individual transcriptome samples from the P1K public data set (45). (D) Comparison of the CpxR regulon determined in the presence of gentamicin with previously known regulons. (E) CpxR binding to promoter regions of genes, as identified through ChIP-Seq analysis.

To gain a deeper understanding of the relationship between electron transport systems, membrane potential, and the cellular response to gentamicin, we analyzed transcriptome changes under anaerobic and gentamicin conditions (Table S4). These transcriptomic data were projected onto the predetermined independently modulated gene set (iModulon), derived from over a thousand *E. coli* K-12 transcriptome profiles (45). Among the iModulons inferred across gentamicin and anaerobic conditions, we found a specific regulation of the ArcA and CpxR regulons (Fig. 5A; Table S5).

The ArcAB two-component system (TCS) moderates redox in *E. coli*, initiating the aerobic-to-anaerobic transition by inducing quinone adaptation and repressing respiration (46). The CpxAR TCS, which senses misfolded proteins and envelope stress, controls various cellular functions (47). Previous studies have emphasized the importance of bacterial respiration and membrane homeostasis in aminoglycoside susceptibility. Bactericidal efficacy has been shown to correlate with metabolic activity and respiratory flux (48), and aminoglycoside-induced mistranslation and membrane damage have been linked to oxidative stress and hydroxyl radical formation (12). Both Cpx and Arc systems have been implicated in sensing envelope and redox stress, respectively, although the regulatory mechanisms linking them to aminoglycoside response have remained incompletely understood.

To experimentally assess the essential roles of ArcAB and CpxR in gentamicin resistance, we examined the growth of *cpx* and *arc* deletion mutants under gentamicin (Fig. 5B). Deletion of *arcA*, *arcB*, or *cpxR* significantly increased sensitivity to gentamicin, whereas *cpxA* deletion had little effect (Fig. 5B). This finding contrasts with earlier interpretations assuming that *cpxA* deletion would impair Cpx signaling (12). Instead, previous studies have shown that *cpxA* deletion can lead to CpxR hyperactivation due to loss of phosphatase activity of CpxA (49). This demonstrates that both ArcA and CpxR are required for gentamicin resistance.

Interestingly, previous studies indicated that the Cpx response not only supports envelope protein homeostasis but also regulates respiratory chain components. The Cpx system has been shown to promote turnover of respiratory complexes such as NADH dehydrogenase I and succinate dehydrogenase (50) and to mediate transcriptional changes that impact broader responses (51). Furthermore, there has been evidence of potential crosstalk between the ArcA response regulator and the CpxA sensor kinase during gentamicin exposure (52).

Transcriptomic analysis further revealed that ArcA iModulon activity was highest in the *arcA* knockout strain (Fig. 5C and Table S6), consistent with ArcA's role as a repressor of aerobic metabolism. Under anaerobic conditions, ArcA iModulon activity was minimal, in line with public data sets (P1K anaerobic), while LB control samples showed intermediate activity between anaerobic and *arcA* knockout (P1K Δ*arcA*) samples. According to the P1K data set, the CpxR iModulon is activated under ethanol stress but becomes inactive in the *cpxR* knockout mutant (Fig. 5C). Under gentamicin treatment, CpxR iModulon activity surpasses that of ethanol stress, indicating a more intense stress response (Fig. 5C). Notably, ArcA activity was almost completely suppressed under gentamicin exposure, as the ArcA iModulon activity closely matched that of the P1K Δ*arcA* strain, indicating de-repression of the response (Fig. 5C). Since ArcA is a repressor, its activity is inversely correlated with the activity of the ArcA iModulon.

To further investigate the interplay between ArcA and CpxR, we employed chromatin immunoprecipitation sequencing (ChIP-Seq) on CpxR. Under gentamicin, CpxR bound to 933 positions in the genome, compared to just 51 under anaerobic conditions and 13 in LB control (Table S7). These results suggest that CpxR serves as a global regulator under gentamicin, modulating 1,611 genes, far more than the previously reported 72 or 260 regulons (Fig. 5D) (53–55). Transcriptomic analysis showed that a substantial portion of gentamicin-induced DEGs (929 out of 2,348 DEGs) were directly associated with CpxR binding, particularly stress-response genes such as *degP*, *cpxP*, and sigma factors *rpoE* and *rpoH*, which are also directly regulated by *arcA* (Fig. 5E) (12). Importantly, CpxR also bound to the regulatory regions of respiratory complexes, including NADH dehydrogenase (NDH1; encoded by *nuoABCEFGHIJKLMN*), cytochrome *bo_3_* oxidoreductase (Cyt*_bo_*; encoded by *cyoABCD*), and Cyt*_bd_*_-I_ (Cyt*_bd_*_-I_; encoded by *cydABXH*) under gentamicin (Fig. 5E).

**Fig 6:**
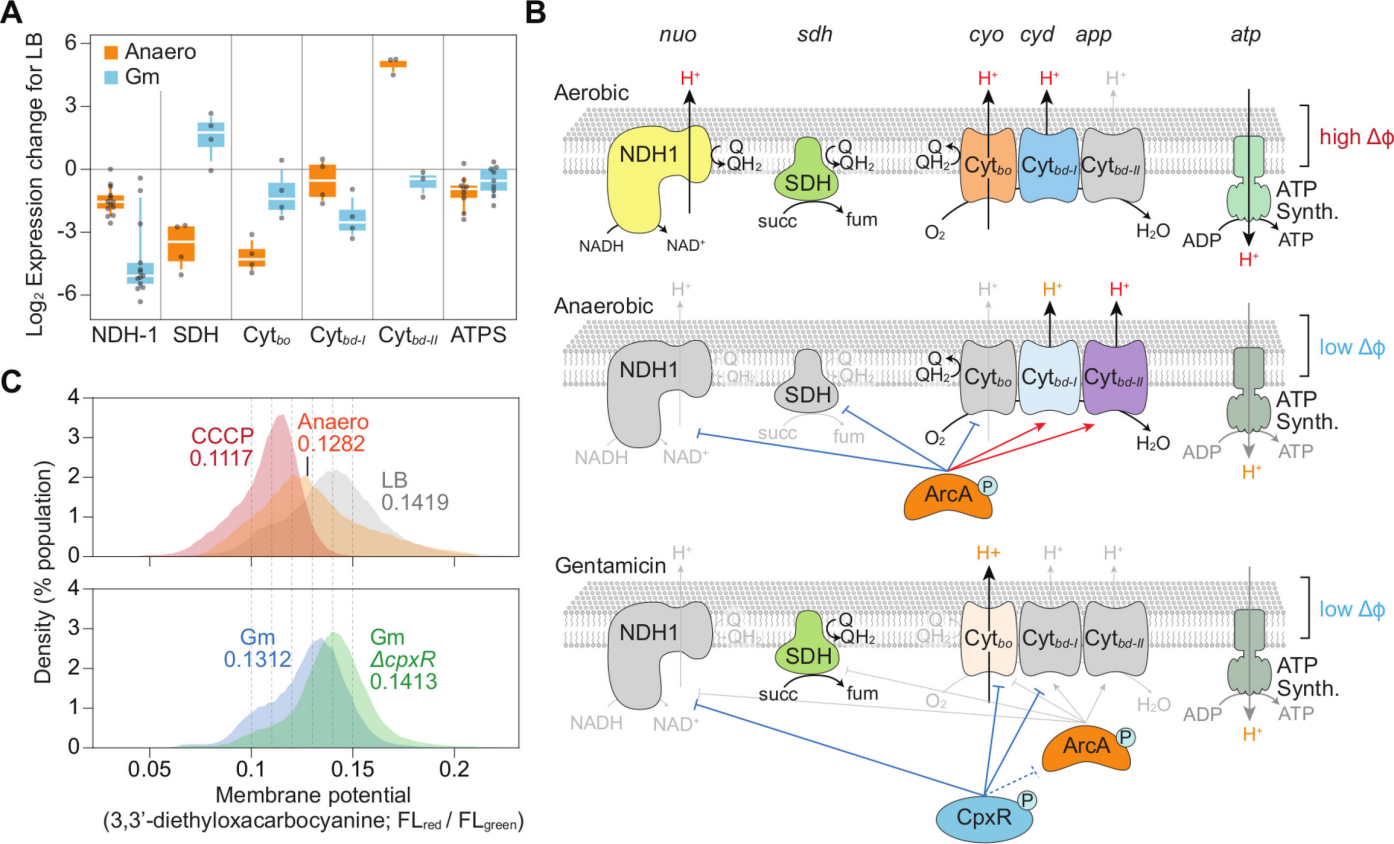
Respiratory adaptation in *E. coli,* conditionally regulated by CpxR and ArcA. (A) RNA-seq analysis showing changes in the expression of respiratory complexes under anaerobic and gentamicin-treated conditions, relative to aerobic LB conditions. Box plots depict the first and the third quartiles (box limits), 10th and 90th percentiles (whiskers), and medians (center lines). Individual dots represent the expression of specific genes. (B) A proposed transcriptional model for the respiratory system by ArcA and CpxR under aerobic, anaerobic, and gentamicin-treated conditions. Symbols: Δϕ: membrane potential. NDH-1: NADH-quinone oxidoreductase. SDH: succinate dehydrogenase. Cyt: cytochrome. ATP Synth: ATP synthase. (C) Flow cytometry histograms showing the membrane potential of *E. coli* K-12 BW25113 and the *cpxR* knockout strain under aerobic, anaerobic, CCCP, and gentamicin-treated conditions. CCCP, which dissipates the proton gradient, is used as an uncoupling control. Numbers indicate the median membrane potential of the populations.

Under aerobic conditions, the transcription levels of the *nuo*, *sdh*, *cyo*, and *cyd*, and *atp* gene clusters remained high (Table S4). Under anaerobic conditions, NDH1, succinate dehydrogenase (SDH), and Cyt*_bo_* appear to be repressed by ArcA, consistent with previous findings (Fig. 6A and B) (56, 57). However, Cyt*_bd_*_-I_ showed only a slight reduction in expression. In contrast, the alternative cytochrome *bd*-II (Cyt_*bd*-II_; encoded by *appCBX*), which has higher oxygen affinity and different quinone affinity, exhibited significantly increased expression, specifically under anaerobic conditions (Fig. 6A and B).

From ChIP-Seq and RNA-seq results, we observed that CpxR regulates the expression of genes involved in the respiratory systems in a way that dominates or modulates the regulatory effects of ArcA. Specifically, CpxR induces the repression of the *nuo*, *cyo*, and *cyd* respiration systems under gentamicin stress. Although it is not fully clear whether this repression occurs independently of ArcA, it appears that CpxR may have a regulatory influence that supersedes that of ArcA in this context. This regulation leads to the repression of all respiratory complexes contributing to the proton gradient, such as NDH1, Cyt*_bo_*, and Cyt*_bd_*_-I_ (Fig. 6A and B). Although CpxR bound to the regulatory region of *arcA* under gentamicin (Fig. 5E), *arcA* expression showed only a slight decrease (−6.3%; Table S4), suggesting that CpxR directly regulates the repression of *nuo*, *cyo*, and *cyd* respiration systems independent of ArcA (see also bindings of CpxR in Fig. 5E). While *arcA* may affect the inhibition of *nuo* and *cyo*, it did not affect the activation of *cyd* and *app*, as seen under anaerobic conditions.

This indicates that CpxR plays a pivotal role in controlling membrane potential by repressing the respiration system under gentamicin stress. To further investigate how CpxR affects membrane potential, we examined the membrane potential under LB, anaerobic, and gentamicin conditions, including the use of CCCP, a well-known PMF blocker (Fig. 6C). Flow cytometry analysis revealed that CpxR mediated the collapse of membrane potential in response to gentamicin, as this collapse was absent in the *cpxR* knockout strain (Fig. 6C). Collectively, these findings suggest that, under gentamicin stress, CpxR not only coordinates the protein stress response but also prevents the formation of an electrochemical gradient. The dissipation of this gradient by CpxR could potentially play a role in limiting gentamicin influx.

## DISCUSSION

In this study, we investigated the fitness change of *E. coli* under gentamicin and anaerobic conditions using genome-wide CRISPRi screening. This high-resolution approach enabled us to estimate the essentiality of each gene with high accuracy, without the need for conventional gene knockouts or knockdowns. Notably, the essential genes identified in previous knockout studies closely aligned with those found through our CRISPRi screening (58). This demonstrates that the CRISPRi method offers a rapid and reliable way to study gene regulation, cellular mechanism, metabolism, and more, by monitoring fitness responses to various stimuli.

Our primary focus was on understanding antibiotic responses and cellular regulation under gentamicin exposure. CRISPRi screening showed that gentamicin increased the essentiality of not only ribosomes but also ribosome-associated proteins and ribosomal RNA modification proteins. In addition, specific ribosomal modification enzymes, such as those involved in acetylation, adenylation, or phosphorylation, appeared to play significant roles following gentamicin treatment. These modifications may inactivate gentamicin by altering its binding sites on the ribosome. Moreover, the essentiality of rRNA methyltransferase genes like *rsmD* and *rlmB* increased significantly under gentamicin, suggesting a potential role for rRNA modification enzymes in antibiotic defense.

Gentamicin also impacted toxin-antitoxin systems, which are involved in membrane damage and translation regulation. The increased essentiality of antitoxin genes under gentamicin implies that regulating antitoxins may help compensate for translation inhibition and membrane damage caused by toxins. Overall, our investigation into cellular responses offers insights into the molecular mechanisms underlying antibiotic activity and resistance.

Aminoglycosides, including gentamicin, enter cells through a PMF across the inner membrane (59) and require a certain level of membrane potential (43). This membrane potential is maintained by the respiratory electron transport system, which relies on cofactors and electron carriers such as quinones and hemes. Interestingly, knockdown of quinone and heme synthesis genes provided a notable fitness advantage under gentamicin treatment, consistent with the knockout experiments (Fig. 4C). This observation also explains resistance to gentamicin under anaerobic conditions.

Transcriptomic analysis using iModulons revealed that the response regulator CpxR is involved in the gentamicin response, as observed by the severe growth defect in the *cpxR* knockout strain under gentamicin. Previous reports suggested crosstalk between the CpxAR and ArcAB TCS during gentamicin exposure, but the precise regulatory mechanism remains unresolved (12, 49, 52). A genome-wide binding assay of CpxR under gentamicin revealed that CpxR binds to promoters of genes related not only to proteases and chaperones but also to respiration and directly to *arcA*. This result suggests that CpxR plays a global regulatory role in *E. coli,* regulating protein quality and respiration. While the detailed molecular relationship between CpxR and respiration requires further investigation, it is likely that CpxR-mediated inhibition of the respiratory system helps protect vulnerable membrane-bound respiratory proteins from gentamicin-induced damage. Furthermore, substantial evidence highlights the role of Cpx, or its upstream signaling partner NlpE, in the pathogenesis of *E. coli* (60) and other gram-negative pathogens (61–63). Cpx may also contribute to antibiotic resistance in several gram-negative bacteria (64), suggesting that the CpxR-mediated antibiotic response could be a widespread mechanism among gram-negative bacteria.

In summary, genome-wide CRISPRi screening provides a rapid and robust approach for exploring fundamental processes in *E. coli*. This study offers new insights into the underlying molecular mechanisms of antibiotic action and the genetic basis of bacterial responses to antibiotics. The data generated from this screening could be a valuable data set for identifying potential targets for novel antibiotics or sensitizers and offers a foundation for further in-depth studies into transcriptional regulatory networks and resistance mechanisms with potential clinical implications.

## MATERIALS AND METHODS

### Cells and plasmids

For library construction, *E. coli* Lucigen's Endura competent cells (Lucigen, WI, USA) were used, while *E. coli* K-12 MG1655 was employed for CRISPRi library screening. The pdCas9 plasmid (Addgene, cat. #46569) and the pgRNA-bacteria plasmid (Addgene, cat. #44251) targeting *mrfp* were used for the expression of dCas9 and sgRNA, respectively. The pgRNA-bacteria plasmid served as a template for generating new sgRNAs. To test growth defects, we used single knockout strains from the KEIO collection (58), including *fre*, *ubiX*, *ubiC*, *hemX*, *bhsA, cpxA, cpxR, arcA,* and *arcB*, with *E. coli* K-12 BW25113 as the wild-type control. Growth profiles were monitored using a Synergy H1 microplate reader (BioTek, VT, USA). For ChIP-Seq, a c-myc tag was added to the C-terminus of *cpxR* in *E. coli* K-12 MG1655 as previously described (65). Primer sequences are summarized in Table S8.

### sgRNA library design and construction

The CRISPRi sgRNA library for *E. coli* K-12 MG1655 genome was designed to target genic regions using 23-mer sequences that start with the protospacer adjacent motif (PAM) sequence NGG (66). sgRNA candidates were selected for all coding sequences (CDS) in the reference genome (RefSeq NC_000913.3) and fragmented every 100 nt. Through BLASTN analysis, multiple-aligned sgRNAs were excluded from sgRNA candidates to avoid off-target binding (67). An in-house Python script was used to match sgRNA to these fragments, selecting the first sequence from each 100 nt window or, if no match was found, using the last sgRNA from the previous fragment. This resulted in a pool of 39,591 double-stranded oligos, each consisting of a 20 nt spacer sequence targeting the genome, flanked by sequences at both 5′ and 3′ ends. The oligo pool was synthesized by CustomArray Inc. (Bothell, WA, USA), using a 5′-GCTCAGTCCTAGGTATAATACTAGTA-20 nt spacer-GTTTTAGAGCTAGAAATAGCAAGTTAAAATAAG-3′ (Table S1). To minimize the deviation in transcription activity due to the transcription start nucleotide, we set the nucleotide upstream of the spacer sequence to “A.”

The CRISPRi library construction was based on a modified version of a previously described method (68). The synthetic double-stranded sgRNA pool was extended and amplified by PCR using the primers sgRNA-exF and sgRNA-exR (Table S8). The plasmid backbone was also amplified by inverse PCR from the pgRNA-bacteria plasmid DNA (Addgene, cat. # 44251) using primers pgRNA-F and pgRNA-R (Table S8) and further treated with *Dpn*I to remove the template plasmid. Both the amplified sgRNA pool and plasmid backbone were purified by gel electrophoresis using a Qiagen gel extraction kit (Qiagen, Hilden, Germany).

Next, 40 ng of amplified sgRNA library products (200 bp) was assembled into 100 ng of plasmid backbone (2,435 bp) in a 40 µL reaction using the NEBuilder HiFi DNA Assembly Kit (NEB, MA, USA). Following assembly, 0.5 µL of each reaction was transformed into Endura ElectroCompetent Cells using a Gene Pulser II Electroporation System (BioRad, Hercules, USA). Transformants were plated on 87 LB agar plates (25 cm × 25 cm) containing ampicillin (100 µg/mL) and incubated. We obtained a total of 0.8 × 10^7^ colonies, ensuring 200 × coverage of the designed sgRNAs. Colonies were scraped from each plate, and residual cells were collected by washing the plates with 50 mL of phosphate-buffered saline (PBS) and pooling with the collected colonies. The pooled cells were centrifuged at 4,000 × *g* for 10 min at 4°C to remove the supernatant. The mass of the cell pellet was measured and adjusted to a concentration of 0.25 g/mL in PBS. From the combined cells (80 mg), the plasmid pool was extracted using the PureYield Plasmid Midiprep System (Promega, WI, USA). The plasmid pool was further purified using 1% agarose gel to remove nonspecific DNA and contaminating gDNA. Aliquots of 50 µL (100 µg/mL) of the plasmid pool were stored at −80°C with glycerol for later use.

### sgRNA library preparation for CRISPRi screening

For CRISPRi screening, we transformed the sgRNA library plasmids (100 ng) into *E. coli* K-12 MG1655 competent cells that already contained the pdCas9 plasmid by electroporation. The transformed cells were then spread on LB plates pre-warmed to 30°C, containing both chloramphenicol (35 µg/mL) and ampicillin (100 µg/mL) to ensure the selection of cells containing both plasmids. After 14 h of incubation at 30°C, approximately 2.0 × 10^6^ colonies were obtained from 24 plates. Colonies from each plate were combined by weight, similarly to the method used during sgRNA library construction. The resuspended cells were aliquoted with glycerol and stored at −80°C for later use.

For liquid culture, the CRISPRi library cells were precultured in 50 mL LB with chloramphenicol and ampicillin until the optical density at 600 nm (OD_600_) reached 1.0. Then, these cells were inoculated into fresh LB media at an initial OD_600_ of 0.05, with and without gentamicin (1 µg/mL), while maintaining chloramphenicol and ampicillin. Aerobic cultures were grown at 37°C with shaking at 250 rpm until the OD_600_ reached 1.68 in LB and 1.8 in LB with gentamicin (Fig. S1A).

For anaerobic cultures, LB medium was stored in a nitrogen-filled chamber immediately after autoclaving to maintain anaerobic conditions. Then, 60 mL of LB was transferred into bottles sealed with butyl rubber stoppers, and nitrogen gas was added to the headspace at a pressure of 100 kPa. Pre-cultured CRISPRi library cells were inoculated into these anaerobic cultures using a syringe at an initial OD_600_ of 0.05. The cultures were grown with shaking at 37°C for 9 h until the OD_600_ reached 0.6 (Fig. S1B).

### sgRNA library sequencing

CRISPRi library cells were cultured, then centrifuged at 4,000 × *g* for 10 min at 4°C. The sgRNA plasmid pools were purified using the PureYield Plasmid Midiprep System. To construct the sequencing library, we amplified sgRNA pool sequences from 50 ng plasmids with TruSeq-based primers containing index and adapter sequences. Amplification was carried out for 18–20 cycles, reaching a semi-plateau (Table S8), and monitored with real-time PCR using SYBR Green. The amplified products were separated by agarose gel electrophoresis and concentrated with a MinElute Gel Extraction Kit. The purified libraries were quantified using a Qubit 2.0 fluorometer (Invitrogen) and sequenced to single-end mode on an Illumina HiSeq 2500 system, with a read length of 150 bp (Illumina, CA, USA).

### RNA sequencing (RNA-Seq) and analysis

*E. coli* K-12 MG1655 was cultured in LB, LB with 1 µg/mL gentamicin, and LB under anaerobic conditions, following the CRISPRi screening protocol. The experiment was conducted in triplicate. Total RNA was extracted using the RNAsnap method (69), and then precipitated with ethanol. mRNA was purified after removing rRNA using the Ribo-zero magnetic kit for bacteria (Epicentre, WI, USA), following the manufacturer's instructions. The sequencing library was then constructed with the TruSeq Stranded mRNA Sample Prep Kit (Illumina), in accordance with the manufacturer's instructions. The quantified samples were sequenced on an Illumina HiSeq 2500 with a read length of 50 bp. RNA-seq reads were mapped to the *E. coli* MG1655 reference genome (NC_000913.3) using the CLC Genomics Workbench (CLC Bio, Aarhus, Denmark), with length and similarity fractions set to 0.9. Gene expression levels were normalized using the DESeq2 pipeline (70). Genes with more than a 2.0-fold change in expression and a *P*_adj_ value <0.001 were considered differentially expressed genes (DEG). To infer iModulon activities, we projected log_2_-transformed expression levels (centered to LB) onto the pre-existing iModulon structure of *E. coli* K-12 (45) (https://imodulondb.org/dataset.html?organism=e_coli&dataset=precise1k) using the pymodulon python package (https://github.com/SBRG/pymodulon) (71).

### Chromatin immunoprecipitation (ChIP) sequencing

Cultured cells (25 mL) were cross-linked with 1% formaldehyde at room temperature for 30 min. The reaction was stopped by adding 2 mL of 2.5 M glycine. After washing the cells three times with 50 mL of ice-cold Tris-buffered saline, we resuspended the cell pellets in 0.5 mL lysis buffer (50 mM Tris-HCl [pH 7.5], 100 mM NaCl, 1 mM EDTA, 1 µg/mL RNase A, 40 µL protease inhibitor cocktail [50 mg/mL;Sigma-Aldrich], and 1 kU Ready-Lyse lysozyme solution [LGC Biosearch Technologies, Teddington, UK]) and incubated them at 37°C for 30 min. The cells were then mixed with 0.5 mL of 2× IP buffer (100 mM Tris-HCl [pH 7.5], 200 mM NaCl, 2% Triton X-100, and 1 mM EDTA) and incubated on ice for 30 min. The complex was fragmented through 10 cycles of 20 s sonication followed by 30 s of resting using a Sonic Dismembrator Model 500 (Fisher Scientific, Waltham, MA). After centrifugation to remove cell debris, the size distribution of fragmented DNA was confirmed by agarose gel electrophoresis (200–400 bp). Washing buffer I (50 mM Tris-HCl [pH 7.5], 140 mM NaCl, 1% Triton X-100, and 1 mM EDTA) was added to bring the volume to 1.4 mL. A 700 µL aliquot of the sample was used for immunoprecipitation with 15 µL of anti-c-Myc mouse IgG (9E10) (Cat. sc-40) (Santa Cruz Biotechnology, TX, USA) and 2 µL of normal mouse IgG (Cat. sc-2025; Santa Cruz Biotechnology) as a negative control. The reactions were incubated overnight at 4°C with gentle rotation. The antibody-coupled solution was then added to 50 µL of Dynabeads Pan Mouse IgG (Thermo Fisher Scientific, MA, USA) prewashed with 1 mL of ice-cold bead washing buffer (0.5% bovine serum albumin in PBS), and incubation continued at 4°C for 6 h with gentle rotation. The MPC magnet pull-down beads were washed twice with 1 mL of washing buffer I, washing buffer II (50 mM Tris-HCl [pH 7.5], 500 mM NaCl, 1% Triton X-100, and 1 mM EDTA), washing buffer III (10 mM Tris-HCl [pH 8.0], 250 mM LiCl, 1% Triton X-100, and 1 mM EDTA), and TE buffer (10 mM Tris-HCl [pH 8.0] and 1 mM EDTA). We then added 200 µL of elution buffer (50 mM Tris-HCl [pH 8.0], 1% SDS, and 1 mM EDTA) to the washed beads and incubated it with rotation overnight at 65°C for reverse crosslinking. The eluate was incubated at 37°C for 2 h with 1 µL RNase A solution (100 mg/mL) (Qiagen) and further incubated at 55°C for 2 h with 4 µL of proteinase K (20 mg/mL; Invitrogen). The IP DNA was purified using the MinElute PCR Purification Kit (Qiagen) and then modified and amplified using the NEXTflex ChIP-Seq Library Prep Kit for Illumina Sequencing (PerkinElmer, MA, USA). Sequencing libraries were quantified using a Qubit dsDNA HS Assay Kit (Thermo Fisher Scientific) and a Qubit 4.0 fluorometer (Thermo Fisher Scientific). Library quality was assessed using a TapeStation 4150 (Agilent, CA, USA) with a high-sensitivity D1000 ScreenTape (Agilent). The libraries were sequenced with a 50 cycle single-ended reaction on the HiSeq 2500 (Illumina). ChIP-Seq reads were mapped to the *E. coli* MG1655 reference genome (NC_000913.3) using the CLC Genomics Workbench (CLC Bio), with length and similarity fractions set to 0.9. Binding peaks were identified using MACS2 software with default settings (72). Peaks detected in two or more replicates were considered binding events. The operational structure of *E. coli* MG1655 was obtained from the DOOR2.0 data set (73) to determine the affected genes and operons.

### Antibiotic disk diffusion assay

Cells were spread on an LB agar plate, and disks containing 0, 0.1, 1, 2.5, and 10 µM gentamicin were placed on the agar. The plate was incubated at 37°C. For anaerobic cultures, the LB agar plate was prepared in an anaerobic chamber to remove oxygen, and cells were cultivated in an anaerobic incubator. After 16 h of incubation for aerobic cultures and 24 h for anaerobic cultures, the presence of a halo around the disk was observed.

### Membrane potential assay

*E. coli* K-12 BW25113 and BW25113Δ*cpxR* strains were cultured at 37°C until OD_600_ reached 1.0 for aerobic conditions and 0.6 for anaerobic conditions in LB media. If required, 5 µM carbonyl cyanide 3-chlorophenylhydrazone (CCCP) or 1 µg/mL gentamicin was added. The culture was then diluted to 1 × 10^6^ cells/mL with filtered PBS buffer. Membrane potential was assessed using the BacLight Bacterial Membrane Potential Kit (Thermo Fisher Scientific) with the carbocyanine dye DiOC_2_ (3,3′-diethyloxacarbocyanine iodide) according to the manufacturer's instructions. The dye exhibits fluorescence shift from green to red as the membrane potential increases. After 30 min of DiOC_2_ treatment, cells were analyzed with an S3e Cell Sorter (Bio-Rad) using a 488 nm laser. Fluorescence was measured in the green and red channels with 526/48 (FL1) and 593/40 (FL2) emission filters, respectively. Samples were analyzed at a rate of 2,000 events/s with an FSC threshold of 335. A total of 100,000 events were collected and analyzed using FlowJo software v10.2 (FlowJo, OR, USA).

## Data Availability

The high-throughput sequencing data generated in this study have been submitted to the European Nucleotide Archive (ENA; https://www.ebi.ac.uk/ena/browser/home/) under accession number PRJEB33267. Python scripts for designing spacer sequences are available through Github (https://github.com/robinald/EcCRISPRi_design/).

## SUPPLEMENTAL MATERIAL

Supplemental figures msystems.00353-25-s0001.pdf Figures S1–S3.

Table S1 msystems.00353-25-s0002.xlsx A synthetic sgRNA library for targeting *E. coli* K-12 MG1655 coding sequences.

Table S2 msystems.00353-25-s0003.xlsx Normalized sgRNA counts.

Table S3 msystems.00353-25-s0004.xlsx Enrichment ratios.

Table S4 msystems.00353-25-s0005.xlsx Normalized expression levels.

Table S5 msystems.00353-25-s0006.xlsx Inferred iModulon activity matrix.

Table S6 msystems.00353-25-s0007.xlsx CpxR and ArcA iModulon activities.

Table S7 msystems.00353-25-s0008.xlsx Peaks detected by CpxR ChIP-Seq.

Table S8 msystems.00353-25-s0009.xlsx Primers.

ASM does not own the copyrights to Supplemental Material that may be linked to, or accessed through, an article. The authors have granted ASM a non-exclusive, world-wide license to publish the Supplemental Material files. Please contact the corresponding author directly for reuse.

## References

[1] Storici F, Lewis LK, Resnick MA. 2001. In vivo site-directed mutagenesis using oligonucleotides. Nat Biotechnol 19:773–776. doi:10.1038/90837 11479573

[2] van Opijnen T, Bodi KL, Camilli A. 2009. Tn-seq: high-throughput parallel sequencing for fitness and genetic interaction studies in microorganisms. Nat Methods 6:767–772. doi:10.1038/nmeth.1377 19767758 PMC2957483

[3] Gilbert LA, Horlbeck MA, Adamson B, Villalta JE, Chen Y, Whitehead EH, Guimaraes C, Panning B, Ploegh HL, Bassik MC, Qi LS, Kampmann M, Weissman JS. 2014. Genome-scale CRISPR-mediated control of gene repression and activation. Cell 159:647–661. doi:10.1016/j.cell.2014.09.029 25307932 PMC4253859

[4] Qi LS, Larson MH, Gilbert LA, Doudna JA, Weissman JS, Arkin AP, Lim WA. 2013. Repurposing CRISPR as an RNA-guided platform for sequence-specific control of gene expression. Cell 152:1173–1183. doi:10.1016/j.cell.2013.02.022 23452860 PMC3664290

[5] Wang T, Guan C, Guo J, Liu B, Wu Y, Xie Z, Zhang C, Xing XH. 2018. Pooled CRISPR interference screening enables genome-scale functional genomics study in bacteria with superior performance. Nat Commun 9:2475. doi:10.1038/s41467-018-04899-x 29946130 PMC6018678

[6] Evers B, Jastrzebski K, Heijmans JPM, Grernrum W, Beijersbergen RL, Bernards R. 2016. CRISPR knockout screening outperforms shRNA and CRISPRi in identifying essential genes. Nat Biotechnol 34:631–633. doi:10.1038/nbt.3536 27111720

[7] Chaves BJ, Tadi P. 2024. Gentamicin. StatPearls, Treasure Island, FL.

[8] Krause KM, Serio AW, Kane TR, Connolly LE. 2016. Aminoglycosides: an overview. Cold Spring Harb Perspect Med 6:a027029. doi:10.1101/cshperspect.a027029 27252397 PMC4888811

[9] Davies J, Gorini L, Davis BD. 1965. Misreading of RNA codewords induced by aminoglycoside antibiotics. Mol Pharmacol 1:93–106. doi:10.1016/S0026-895X(25)14722-6 4284262

[10] Magnet S, Blanchard JS. 2005. Molecular insights into aminoglycoside action and resistance. Chem Rev 105:477–498. doi:10.1021/cr0301088 15700953

[11] Davis BD, Chen LL, Tai PC. 1986. Misread protein creates membrane channels: an essential step in the bactericidal action of aminoglycosides. Proc Natl Acad Sci USA 83:6164–6168. doi:10.1073/pnas.83.16.6164 2426712 PMC386460

[12] Kohanski MA, Dwyer DJ, Wierzbowski J, Cottarel G, Collins JJ. 2008. Mistranslation of membrane proteins and two-component system activation trigger antibiotic-mediated cell death. Cell 135:679–690. doi:10.1016/j.cell.2008.09.038 19013277 PMC2684502

[13] Lee MD, Sanchez S, Zimmer M, Idris U, Berrang ME, McDermott PF. 2002. Class 1 integron-associated tobramycin-gentamicin resistance in Campylobacter jejuni isolated from the broiler chicken house environment. Antimicrob Agents Chemother 46:3660–3664. doi:10.1128/AAC.46.11.3660-3664.2002 12384387 PMC128761

[14] Nirdnoy W, Mason CJ, Guerry P. 2005. Mosaic structure of a multiple-drug-resistant, conjugative plasmid from Campylobacter jejuni. Antimicrob Agents Chemother 49:2454–2459. doi:10.1128/AAC.49.6.2454-2459.2005 15917546 PMC1140535

[15] Peterson E, Kaur P. 2018. Antibiotic resistance mechanisms in bacteria: relationships between resistance determinants of antibiotic producers, environmental bacteria, and clinical pathogens. Front Microbiol 9:2928. doi:10.3389/fmicb.2018.02928 30555448 PMC6283892

[16] Bryan LE, Van Den Elzen HM. 1975. Gentamicin accumulation by sensitive strains of Escherichia coli and Pseudomonas aeruginosa. J Antibiot (Tokyo) 28:696–703. doi:10.7164/antibiotics.28.696 810469

[17] Hoffman LR, D’Argenio DA, MacCoss MJ, Zhang Z, Jones RA, Miller SI. 2005. Aminoglycoside antibiotics induce bacterial biofilm formation. Nature 436:1171–1175. doi:10.1038/nature03912 16121184

[18] Bylund GO, Wipemo LC, Lundberg LA, Wikström PM. 1998. RimM and RbfA are essential for efficient processing of 16S rRNA in Escherichia coli. J Bacteriol 180:73–82. doi:10.1128/JB.180.1.73-82.1998 9422595 PMC106851

[19] Kazakov T, Kuznedelov K, Semenova E, Mukhamedyarov D, Datsenko KA, Metlitskaya A, Vondenhoff GH, Tikhonov A, Agarwal V, Nair S, Van Aerschot A, Severinov K. 2014. The RimL transacetylase provides resistance to translation inhibitor microcin C. J Bacteriol 196:3377–3385. doi:10.1128/JB.01584-14 25002546 PMC4187662

[20] Shakil S, Khan R, Zarrilli R, Khan AU. 2008. Aminoglycosides versus bacteria - a description of the action, resistance mechanism, and nosocomial battleground. J Biomed Sci 15:5–14. doi:10.1007/s11373-007-9194-y 17657587

[21] Jaffé A, Ogura T, Hiraga S. 1985. Effects of the CCD function of the F plasmid on bacterial growth. J Bacteriol 163:841–849. doi:10.1128/jb.163.3.841-849.1985 3897195 PMC219208

[22] Page R, Peti W. 2016. Toxin-antitoxin systems in bacterial growth arrest and persistence. Nat Chem Biol 12:208–214. doi:10.1038/nchembio.2044 26991085

[23] Cheng HY, Soo VWC, Islam S, McAnulty MJ, Benedik MJ, Wood TK. 2014. Toxin GhoT of the GhoT/GhoS toxin/antitoxin system damages the cell membrane to reduce adenosine triphosphate and to reduce growth under stress. Environ Microbiol 16:1741–1754. doi:10.1111/1462-2920.12373 24373067

[24] Wang X, Lord DM, Cheng HY, Osbourne DO, Hong SH, Sanchez-Torres V, Quiroga C, Zheng K, Herrmann T, Peti W, Benedik MJ, Page R, Wood TK. 2012. A new type V toxin-antitoxin system where mRNA for toxin GhoT is cleaved by antitoxin GhoS. Nat Chem Biol 8:855–861. doi:10.1038/nchembio.1062 22941047 PMC3514572

[25] Pedersen K, Zavialov AV, Pavlov MY, Elf J, Gerdes K, Ehrenberg M. 2003. The bacterial toxin RelE displays codon-specific cleavage of mRNAs in the ribosomal A site. Cell 112:131–140. doi:10.1016/s0092-8674(02)01248-5 12526800

[26] Zhang Y, Yamaguchi Y, Inouye M. 2009. Characterization of YafO, an Escherichia coli toxin. J Biol Chem 284:25522–25531. doi:10.1074/jbc.M109.036624 19617347 PMC2757953

[27] Sat B, Hazan R, Fisher T, Khaner H, Glaser G, Engelberg-Kulka H. 2001. Programmed cell death in Escherichia coli: some antibiotics can trigger mazEF lethality. J Bacteriol 183:2041–2045. doi:10.1128/JB.183.6.2041-2045.2001 11222603 PMC95100

[28] Christensen-Dalsgaard M, Jørgensen MG, Gerdes K. 2010. Three new RelE-homologous mRNA interferases of Escherichia coli differentially induced by environmental stresses. Mol Microbiol 75:333–348. doi:10.1111/j.1365-2958.2009.06969.x 19943910 PMC2814082

[29] Brown BL, Grigoriu S, Kim Y, Arruda JM, Davenport A, Wood TK, Peti W, Page R. 2009. Three dimensional structure of the MqsR:MqsA complex: a novel TA pair comprised of a toxin homologous to RelE and an antitoxin with unique properties. PLoS Pathog 5:e1000706. doi:10.1371/journal.ppat.1000706 20041169 PMC2791442

[30] Hu Y, Benedik MJ, Wood TK. 2012. Antitoxin DinJ influences the general stress response through transcript stabilizer CspE. Environ Microbiol 14:669–679. doi:10.1111/j.1462-2920.2011.02618.x 22026739 PMC7261204

[31] Weiner L, Brissette JL, Model P. 1991. Stress-induced expression of the Escherichia coli phage shock protein operon is dependent on sigma 54 and modulated by positive and negative feedback mechanisms. Genes Dev 5:1912–1923. doi:10.1101/gad.5.10.1912 1717346

[32] Bos MP, Robert V, Tommassen J. 2007. Biogenesis of the gram-negative bacterial outer membrane. Annu Rev Microbiol 61:191–214. doi:10.1146/annurev.micro.61.080706.093245 17506684

[33] Sugawara E, Nikaido H. 1992. Pore-forming activity of OmpA protein of Escherichia coli. J Biol Chem 267:2507–2511. doi:10.1016/S0021-9258(18)45908-X 1370823

[34] Smani Y, Fàbrega A, Roca I, Sánchez-Encinales V, Vila J, Pachón J. 2014. Role of OmpA in the multidrug resistance phenotype of Acinetobacter baumannii. Antimicrob Agents Chemother 58:1806–1808. doi:10.1128/AAC.02101-13 24379205 PMC3957889

[35] Overly Cottom C, Stephenson R, Wilson L, Noinaj N. 2023. Targeting BAM for novel therapeutics against pathogenic gram-negative bacteria. Antibiotics (Basel) 12:679. doi:10.3390/antibiotics12040679 37107041 PMC10135246

[36] Anes J, McCusker MP, Fanning S, Martins M. 2015. The ins and outs of RND efflux pumps in Escherichia coli. Front Microbiol 6:587. doi:10.3389/fmicb.2015.00587 26113845 PMC4462101

[37] Du D, Wang-Kan X, Neuberger A, van Veen HW, Pos KM, Piddock LJV, Luisi BF. 2018. Multidrug efflux pumps: structure, function and regulation. Nat Rev Microbiol 16:523–539. doi:10.1038/s41579-018-0048-6 30002505

[38] Morona R, Manning PA, Reeves P. 1983. Identification and characterization of the TolC protein, an outer membrane protein from Escherichia coli. J Bacteriol 153:693–699. doi:10.1128/jb.153.2.693-699.1983 6337123 PMC221686

[39] Hancock RE, Farmer SW, Li ZS, Poole K. 1991. Interaction of aminoglycosides with the outer membranes and purified lipopolysaccharide and OmpF porin of Escherichia coli. Antimicrob Agents Chemother 35:1309–1314. doi:10.1128/AAC.35.7.1309 1656859 PMC245163

[40] Jana S, Deb JK. 2006. Molecular understanding of aminoglycoside action and resistance. Appl Microbiol Biotechnol 70:140–150. doi:10.1007/s00253-005-0279-0 16391922

[41] Tatusov RL, Galperin MY, Natale DA, Koonin EV. 2000. The COG database: a tool for genome-scale analysis of protein functions and evolution. Nucleic Acids Res 28:33–36. doi:10.1093/nar/28.1.33 10592175 PMC102395

[42] Alexander K, Young IG. 1978. Alternative hydroxylases for the aerobic and anaerobic biosynthesis of ubiquinone in Escherichia coli. Biochemistry 17:4750–4755. doi:10.1021/bi00615a024 365223

[43] Bryan LE, Kwan S. 1983. Roles of ribosomal binding, membrane potential, and electron transport in bacterial uptake of streptomycin and gentamicin. Antimicrob Agents Chemother 23:835–845. doi:10.1128/AAC.23.6.835 6351731 PMC184978

[44] Zhang XS, García-Contreras R, Wood TK. 2007. YcfR (BhsA) influences Escherichia coli biofilm formation through stress response and surface hydrophobicity. J Bacteriol 189:3051–3062. doi:10.1128/JB.01832-06 17293424 PMC1855844

[45] Lamoureux CR, Decker KT, Sastry AV, Rychel K, Gao Y, McConn JL, Zielinski DC, Palsson BO. 2023. A multi-scale expression and regulation knowledge base for Escherichia coli. Nucleic Acids Res 51:10176–10193. doi:10.1093/nar/gkad750 37713610 PMC10602906

[46] van Beilen JWA, Hellingwerf KJ. 2016. All three endogenous quinone species of Escherichia coli are involved in controlling the activity of the aerobic/anaerobic response regulator ArcA. Front Microbiol 7:1339. doi:10.3389/fmicb.2016.01339 27656164 PMC5013052

[47] Zhao Z, Xu Y, Jiang B, Qi Q, Tang YJ, Xian M, Wang J, Zhao G. 2022. Systematic identification of CpxRA-regulated genes and their roles in Escherichia coli stress response. mSystems 7:e00419-22. doi:10.1128/msystems.00419-22 36069452 PMC9600279

[48] Lobritz MA, Belenky P, Porter CBM, Gutierrez A, Yang JH, Schwarz EG, Dwyer DJ, Khalil AS, Collins JJ. 2015. Antibiotic efficacy is linked to bacterial cellular respiration. Proc Natl Acad Sci USA 112:8173–8180. doi:10.1073/pnas.1509743112 26100898 PMC4500273

[49] Mahoney TF, Silhavy TJ. 2013. The Cpx stress response confers resistance to some, but not all, bactericidal antibiotics. J Bacteriol 195:1869–1874. doi:10.1128/JB.02197-12 23335416 PMC3624577

[50] Tsviklist V, Guest RL, Raivio TL. 2021. The Cpx stress response regulates turnover of respiratory chain proteins at the inner membrane of Escherichia coli. Front Microbiol 12:732288. doi:10.3389/fmicb.2021.732288 35154019 PMC8831704

[51] Raivio TL, Leblanc SKD, Price NL. 2013. The Escherichia coli Cpx envelope stress response regulates genes of diverse function that impact antibiotic resistance and membrane integrity. J Bacteriol 195:2755–2767. doi:10.1128/JB.00105-13 23564175 PMC3697260

[52] Ćudić E, Surmann K, Panasia G, Hammer E, Hunke S. 2017. The role of the two-component systems Cpx and Arc in protein alterations upon gentamicin treatment in Escherichia coli. BMC Microbiol 17:197. doi:10.1186/s12866-017-1100-9 28923010 PMC5604497

[53] Choudhary KS, Kleinmanns JA, Decker K, Sastry AV, Gao Y, Szubin R, Seif Y, Palsson BO. 2020. Elucidation of regulatory modes for five two-component systems in Escherichia coli reveals novel relationships. mSystems 5:e00980-20. doi:10.1128/mSystems.00980-20 33172971 PMC7657598

[54] Decker KT, Gao Y, Rychel K, Al Bulushi T, Chauhan SM, Kim D, Cho BK, Palsson BO. 2022. proChIPdb: a chromatin immunoprecipitation database for prokaryotic organisms. Nucleic Acids Res 50:D1077–D1084. doi:10.1093/nar/gkab1043 34791440 PMC8728212

[55] Santos-Zavaleta A, Salgado H, Gama-Castro S, Sánchez-Pérez M, Gómez-Romero L, Ledezma-Tejeida D, García-Sotelo JS, Alquicira-Hernández K, Muñiz-Rascado LJ, Peña-Loredo P, Ishida-Gutiérrez C, Velázquez-Ramírez DA, Del Moral-Chávez V, Bonavides-Martínez C, Méndez-Cruz CF, Galagan J, Collado-Vides J. 2019. RegulonDB v 10.5: tackling challenges to unify classic and high throughput knowledge of gene regulation in E. coli K-12. Nucleic Acids Res 47:D212–D220. doi:10.1093/nar/gky1077 30395280 PMC6324031

[56] Iyer MS, Pal A, Srinivasan S, Somvanshi PR, Venkatesh KV. 2021. Global transcriptional regulators fine-tune the translational and metabolic efficiency for optimal growth of Escherichia coli. mSystems 6:e00001-21 doi:10.1128/mSystems.00001-21 33785570 PMC8546960

[57] Park DM, Akhtar MS, Ansari AZ, Landick R, Kiley PJ. 2013. The bacterial response regulator arcA uses a diverse binding site architecture to regulate carbon oxidation globally. PLoS Genet 9:e1003839. doi:10.1371/journal.pgen.1003839 24146625 PMC3798270

[58] Baba T, Ara T, Hasegawa M, Takai Y, Okumura Y, Baba M, Datsenko KA, Tomita M, Wanner BL, Mori H. 2006. Construction of Escherichia coli K-12 in-frame, single-gene knockout mutants: the Keio collection. Mol Syst Biol 2:2006. doi:10.1038/msb4100050 PMC1681482 16738554

[59] Serio AW, Keepers T, Andrews L, Krause KM. 2018. Aminoglycoside revival: review of a historically important class of antimicrobials undergoing rejuvenation. EcoSal Plus 8. doi:10.1128/ecosalplus.ESP-0002-2018 PMC11575671 30447062

[60] Debnath I, Norton JP, Barber AE, Ott EM, Dhakal BK, Kulesus RR, Mulvey MA. 2013. The Cpx stress response system potentiates the fitness and virulence of uropathogenic Escherichia coli. Infect Immun 81:1450–1459. doi:10.1128/IAI.01213-12 23429541 PMC3647988

[61] Carlsson KE, Liu J, Edqvist PJ, Francis MS. 2007. Influence of the Cpx extracytoplasmic-stress-responsive pathway on Yersinia sp.-eukaryotic cell contact. Infect Immun 75:4386–4399. doi:10.1128/IAI.01450-06 17620356 PMC1951158

[62] Siroy A, Cosette P, Seyer D, Lemaître-Guillier C, Vallenet D, Van Dorsselaer A, Boyer-Mariotte S, Jouenne T, Dé E. 2006. Global comparison of the membrane subproteomes between a multidrug-resistant acinetobacter baumannii strain and a reference strain. J Proteome Res 5:3385–3398. doi:10.1021/pr060372s 17137340

[63] Subramaniam S, Müller VS, Hering NA, Mollenkopf H, Becker D, Heroven AK, Dersch P, Pohlmann A, Tedin K, Porwollik S, McClelland M, Meyer TF, Hunke S. 2019. Contribution of the Cpx envelope stress system to metabolism and virulence regulation in Salmonella enterica serovar typhimurium. PLoS One 14:e0211584. doi:10.1371/journal.pone.0211584 30716090 PMC6361445

[64] Tian ZX, Yi XX, Cho A, O’Gara F, Wang YP. 2016. CpxR activates MexAB-OprM efflux pump expression and enhances antibiotic resistance in both laboratory and clinical nalB-type isolates of Pseudomonas aeruginosa. PLoS Pathog 12:e1005932. doi:10.1371/journal.ppat.1005932 27736975 PMC5063474

[65] Cho BK, Knight EM, Palsson BO. 2006. PCR-based tandem epitope tagging system for Escherichia coli genome engineering. Biotechniques 40:67–72. doi:10.2144/000112039 16454042

[66] Shin J, Bae J, Lee H, Kang S, Jin S, Song Y, Cho S, Cho BK. 2023. Genome-wide CRISPRi screen identifies enhanced autolithotrophic phenotypes in acetogenic bacterium Eubacterium limosum. Proc Natl Acad Sci USA 120:e2216244120. doi:10.1073/pnas.2216244120 36716373 PMC9963998

[67] Sanjana NE, Shalem O, Zhang F. 2014. Improved vectors and genome-wide libraries for CRISPR screening. Nat Methods 11:783–784. doi:10.1038/nmeth.3047 25075903 PMC4486245

[68] Shalem O, Sanjana NE, Hartenian E, Shi X, Scott DA, Mikkelson T, Heckl D, Ebert BL, Root DE, Doench JG, Zhang F. 2014. Genome-scale CRISPR-Cas9 knockout screening in human cells. Science 343:84–87. doi:10.1126/science.1247005 24336571 PMC4089965

[69] Stead MB, Agrawal A, Bowden KE, Nasir R, Mohanty BK, Meagher RB, Kushner SR. 2012. RNAsnap. Nucleic Acids Res 40:e156. doi:10.1093/nar/gks680 22821568 PMC3488207

[70] Love MI, Huber W, Anders S. 2014. Moderated estimation of fold change and dispersion for RNA-seq data with DESeq2. Genome Biol 15:550. doi:10.1186/s13059-014-0550-8 25516281 PMC4302049

[71] Sastry AV, Gao Y, Szubin R, Hefner Y, Xu S, Kim D, Choudhary KS, Yang L, King ZA, Palsson BO. 2019. The Escherichia coli transcriptome mostly consists of independently regulated modules. Nat Commun 10:5536. doi:10.1038/s41467-019-13483-w 31797920 PMC6892915

[72] Zhang Y, Liu T, Meyer CA, Eeckhoute J, Johnson DS, Bernstein BE, Nusbaum C, Myers RM, Brown M, Li W, Liu XS. 2008. Model-based analysis of ChIP-Seq (MACS). Genome Biol 9:R137. doi:10.1186/gb-2008-9-9-r137 18798982 PMC2592715

[73] Mao X, Ma Q, Zhou C, Chen X, Zhang H, Yang J, Mao F, Lai W, Xu Y. 2014. DOOR 2.0: presenting operons and their functions through dynamic and integrated views. Nucleic Acids Res 42:D654–D659. doi:10.1093/nar/gkt1048 24214966 PMC3965076

