# Supplementary material for: CRISPRi screening reveals E. coli’s anaerobic-like respiratory adaptations to gentamicin: membrane depolarization by CpxR
Source: mSystems. 2025 Jun 16;10(7):e00353-25. doi: 10.1128/msystems.00353-25 (PMC12282077; doi:10.1128/msystems.00353-25)
Supplement: Supplemental figures — Figures S1–S3. [file msystems.00353-25-s0001.pdf]

Supplemental Material for

CRISPRi screening reveals *E. coli*'s anaerobic-like respiratory adaptations against gentamicin: Membrane depolarization by CpxR regulation

Donghui Choe *et al.*

This file includes:

Supplementary Figures S1-S3

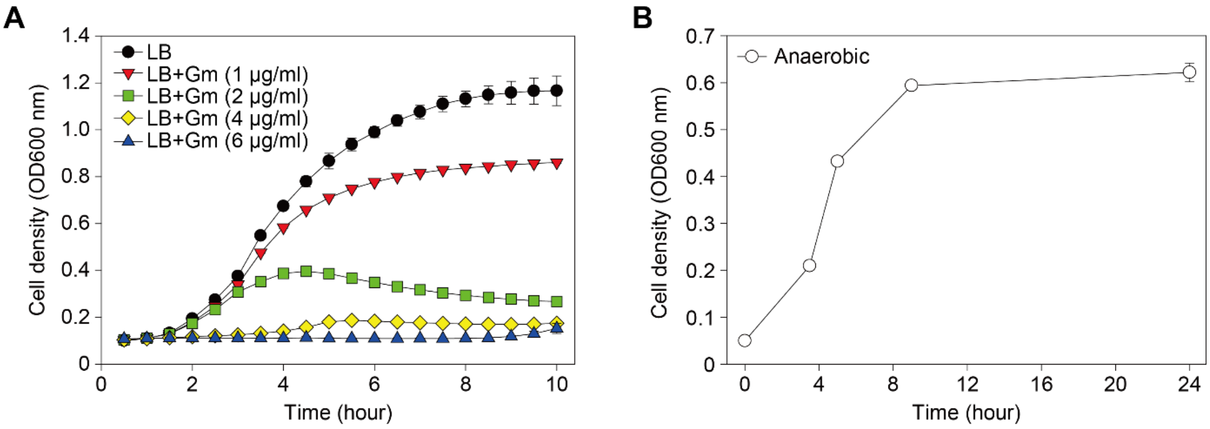

Figure S1. **The growth curve of *E. coli* K-12 MG1655.** (A) Cells were cultured in LB media with gentamicin and their OD_{600} was measured using microplate reader. (B) The cells were cultured under anaerobic condition with LB medium at 37 °C.

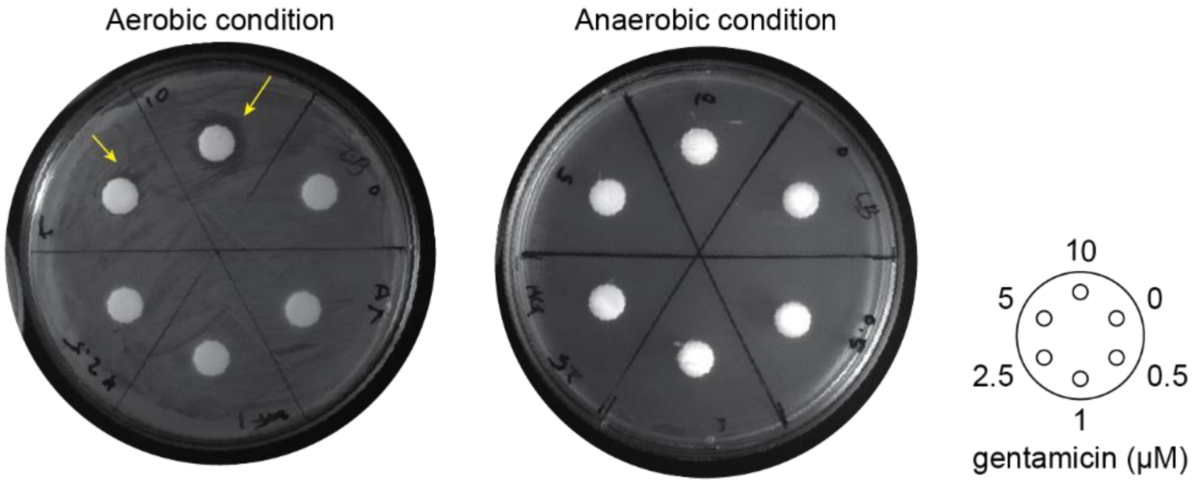

Figure S2. **Disc diffusion assay for gentamycin resistance test.** Gentamicin impregnated filter paper discs of different concentration was placed after spreading corresponding *E. coli* K-12 MG1655 cells on LB agar plate. And the plates were incubated in aerobic chamber for 16 h or anaerobic chamber for 24 h, respectively at 37°C. The yellow arrow in aerobic condition indicates a zone of inhibition by gentamicin.

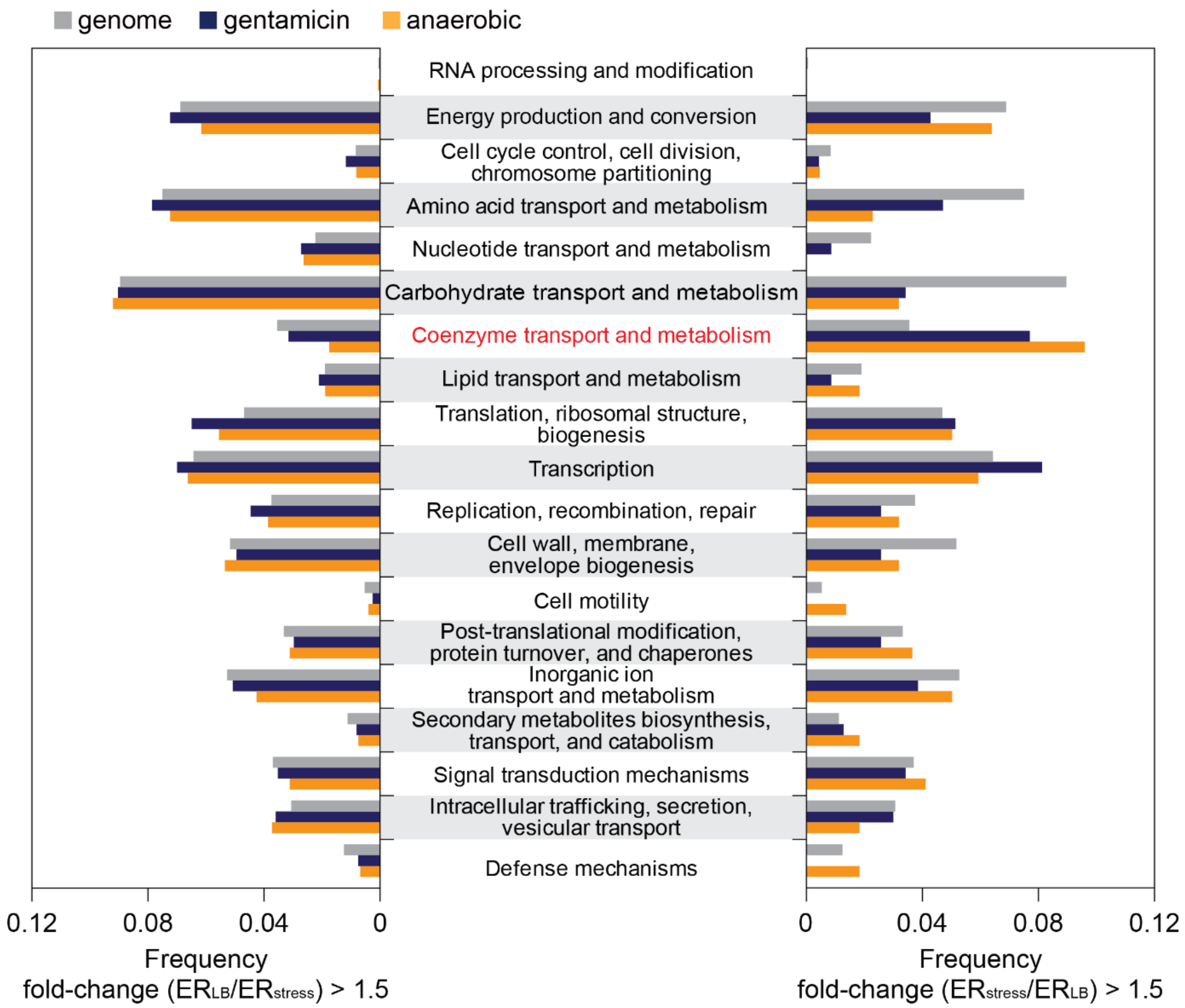

Figure S3. Clusters of Orthologous Groups (COG)-based functional categorization for genes whose ERs were reduced or increased 1.5 times or more under gentamicin (dark blue) or anaerobic condition (orange) than in the LB control. The frequency indicates the proportion of genes for all changed genes. The genome data (gray) represents the entire gene corresponding to the COG classification.
